# Thermoregulatory and cardiovascular responses to creatine, glycerol and alpha lipoic acid in trained cyclists

**DOI:** 10.1186/1550-2783-9-29

**Published:** 2012-06-22

**Authors:** Thelma P Polyviou, Yannis P Pitsiladis, Wu Chean Lee, Takas Pantazis, Catherine Hambly, John R Speakman, Dalia Malkova

**Affiliations:** 1 Institute of Cardiovascular and Medical Sciences, Glasgow, G12 8QQ, United Kingdom; 2 Medical School, College of Medicine, Veterinary and Life Sciences, University of Glasgow, Glasgow, G12 8QQ, United Kingdom; 3 Institute of Biological and Environmental Sciences University of Aberdeen Tillydrone Ave Aberdeen, AB24 2TZ, Scotland, UK

**Keywords:** Creatine, Glycerol, Alpha Lipoic Acid, Thermoregulation, Cardiovascular Response, Water Loading, Exercise

## Abstract

**Background:**

It has been shown that supplementation with creatine (Cr) and glycerol (Gly), when combined with glucose (Glu) necessary for the enhancement of Cr uptake by skeletal muscle, induces significant improvements in thermoregulatory and cardiovascular responses during exercise in the heat.

**Purpose:**

To determine whether Cr/Gly-induced thermoregulatory and cardiovascular responses are maintained when the majority (~75%) of the Glu in the Cr/Gly supplement is replaced with the insulintropic agent alpha lipoic acid (Ala).

**Methods:**

22 healthy endurance trained cyclists were randomly assigned to receive either 20 g/day (4 × 5 g/day) of Cr, 2 g ^.^kg^-1^ BM per day (4 × 0.5 g ^.^kg^-1^ BM per day) of Gly and 150 g/day (4 × 37.5 g/day) of Glu or 20 g/day (4 × 5 g/day) of Cr monohydrate, 2 g ^.^kg^-1^ BM per day (4 × 0.5 g ^.^kg^-1^ BM per day) of Gly (100 g/day (4 × 25 g/day) of Glu and 1000 mg/day (4 × 250 mg/day) of Ala for 7 days for 7 days. Exercise trials were conducted pre- and post-supplementation and involved 40 min of constant-load cycling exercise at 70% O_2_ max by a self-paced 16.1 km time trial at 30°C and 70% relative humidity.

**Results:**

Median and range values of TBW increased significantly by 2.1 (1.3-3.3) L and 1.8 (0.2-4.6) L in Cr/Gly/Glu and Cr/Gly/Glu/Ala groups respectively (*P* = 0.03) and of BM not significantly by 1.8 (0.2-3.0) kg and 1.2 (0.5-2.1) kg in Cr/Gly/Glu and in Cr/Gly/Glu/Ala, respectively (*P* = 0.75). During constant load exercise, heart rate (HR) and core temperature (Tcore) were significantly lower post-supplementation: HR was reduced on average by 3.3 ± 2.1 beats/min and by 4.8 ± 3.3 beats/min (mean ± SD) and Tcore by 0.2 ± 0.1 (mean ± SD) in the Cr/Gly/Glu and Cr/Gly/Glu/Ala, respectively The reduction in HR and Tcore was not significantly different between the supplementation groups.

**Conclusions:**

In comparison to the established hyper hydrating Cr/Gly/Glu supplement, supplement containing Cr/Gly/Ala and decreased amount of Glu provides equal improvements in thermoregulatory and cardiovascular responses during exercise in the heat.

## Background

There is extensive literature advocating the importance of minimizing sweat induced fluid deficits and thus preserve cardiovascular and thermoregulatory function [1]. Although fluid ingestion during exercise is typically practiced in an attempt to maintain water balance, in most cases the rate of sweat loss will be higher than the rate of fluid intake, thus potentially leading to significant dehydration [2]. Similarly, the rapid filtration and excretion by the kidneys of most “excess” water consumed, renders ineffective the approach of hyper hydration prior to exercise via water ingestion alone. Water loading before exercise, using creatine (Cr) combined with glycerol (Gly) has been shown to effectively attenuate cardiovascular and thermoregulatory responses during endurance exercise in the heat [3,4]. Cr inclusion in the hyper hydrating supplement is crucial since Cr retains fluid predominantly in the intracellular fluid compartments [5] while Gly has been shown to have whole-body-hydrating effects [6]. The combination of the two hydrating agents has been shown to have additive effects compared to the sole administration of Cr or Gly as a mean of hyper hydration [3,4].

The typical Cr and Gly hyper hydration strategy requires however, the addition of glucose (Glu) since the release of insulin in response to a rise in blood Glu is needed to stimulate Cr uptake by skeletal muscle [7] and thus is central for hyper hydration. The recommended amount of Glu needed for optimal insulin-mediated Cr uptake is approximately 100 g of Glu per 5 g of Cr ingested and close to the limit of palatability for most individuals [8,9]. It is therefore essential, that an agent, which has insulin-potentiating activity, is found to replace part of the Glu in the Cr and Gly hyper hydrating supplement.

Alpha-lipoic acid (Ala) is a compound known to potentiate Cr uptake under conditions when carbohydrate (CHO) administrated is significantly lower than the recommended doses of 100 g CHO per 5 g of Cr [10]. Ala has indeed been characterized by its pronounced insulin-potentiating activity, with minimal or no effect on plasma Glu levels [11]. Moreover, it has been reported that Ala when ingested with Cr and a small amount of CHO can enhance muscle total Cr content to a greater degree as compared to the ingestion of Cr and CHO alone [10]. Therefore, it can be hypothesized that a hyper hydrating supplement containing Cr, Gly, Ala and decreased amount of Glu compared to the established Cr/Gly/Glu supplement should provide equal improvement in thermoregulatory and cardiovascular responses during exercise in the heat. Therefore, the aim of this study was to examine the effects of the standard Cr/Gly/Glu and the novel Cr/Gly/Glu/Ala supplements consumed for 7 days on thermoregulatory/cardiovascular responses and time trial performance during cycling exercise in the heat in endurance-trained males.

## Methods

### Participants

Twenty-two endurance-trained males (Table 1) took part in the study, which was approved by the local ethics committee and was performed according to the code of ethics of the World Medical Association (Declaration of Helsinki). Participants were in good health and free from any medical condition at the time of testing and regularly took part in strenuous exercise. Eligibility was assessed via an interview and a medical questionnaire. During the interview, the investigator confirmed that participants had not supplemented with Cr in the 6–8 weeks preceding the study; participants were informed of this exclusion criterion at interview and only after their prior Cr supplementation history had been determined. Participants were further questioned about their training practices to confirm all participants were unacclimatized to exercise in the heat at the time of their participation in the study. If participants were considered eligible to take part, they were asked to read and sign a consent form. Prior to giving their written informed consent, participants were fully informed of any risks and discomforts associated with the experiments.

**Table 1:** Physical characteristics of participants

	**Cr/Gly/Glu (*n* = 9)**	**Cr/Gly/Glu/Ala (*n* = 9)**
Age (y)	31 ± 10	32 ± 8
Height (cm)	177 ± 5	182 ± 5
Weight (kg)	71 ± 6	78 ± 8
O_2_max (ml/kg/min)	61 ± 4	59 ± 4
WR_max_ (W)	277 ± 44	242 ± 35

Physical characteristics, maximal oxygen uptake (O_2_max max), maximal work rate (WRmax) of the Cr/Gly/Glu and Cr/Gly/Glu/Ala groups. Data presented as Mean ± SD.

### Study design: Preliminary exercise testing

The lactate threshold (LT), maximal oxygen uptake O_2_ max and maximal work rate (WRmax) were determined using a continuous incremental test to volitional exhaustion on a cycle ergometer (HP Cosmos Cyclus 2 Record-trainer, Nussdorf-Traunstein, Germany) in ambient conditions (i.e., 20–21°C and 30-40% relative humidity). The LT was estimated as work load at which the break-point in the relationship between CO_2_ output (CO_2_) and oxygen consumption (O_2_) occurred and the ventilatory equivalent (E) for O_2_ (E/O_2_) started to increase systematically without a concomitant increase in the ventilator equivalent for CO_2_ (E/CO_2_) [12]. During this test, participants cycled for 5 min at 20 W as a warm up with a gradual increment of 15 W/min thereafter until cadence could no longer be maintained above 50 revolutions/min. Respired volumes and gas concentrations were measured every 15 s using a metabolic cart (Quark CPET b2, Italy, Cosmed). Respired volumes were calibrated with a 3-L syringe using a range of different flow profiles (Hans Rudolph, Kansas City, MO) while respired gas concentrations were calibrated against precision-analyzed gas mixtures.

Following the maximal incremental exercise test, participants reported to the laboratory on three separate occasions (i.e., at least one familiarization trial and two experimental trials). On all occasions, participants were required to cycle for 40 min at a constant pre-determined work rate followed by a 16.1 km self paced time trial at 30°C and 70% relative humidity. On the first occasion, participants underwent a familiarization trial, in order to become familiar with the exercise protocol and experimental procedures. The work rate (WR) at which participants would exercise was calculated by adding 20% of the difference between the WR at the O_2_max and the WR at the LT. In cases when during familiarization trial the desired duration (i.e., 40 min constant load plus 16.1 km time trial) could not be achieved, the WR was decreased to WR at LT for subsequent trials. Prior to the actual experimental trials, familiarization trials were completed until the variability of O_2_ of two consecutive trials was within 5% difference. No subject had to complete a third familiarization trial to achieve less than 5% variability. Following the familiarization trial, participants were matched for body mass (BM) and were randomized in a double-blind fashion to receive Cr/Gly/Glu or Cr/Gly/Glu/Ala. Participants were separated into two groups because of the long washout period associated with Cr [13]. Participants of the the Cr/Gly/Glu group were instructed to ingest 20 g/day (4 × 5 g/day) of Cr monohydrate (Creapure Creatine Monohydrate, Reflex Nutrition Ltd, UK), 2 g^.^kg^-1^ BM per day (4 × 0.5 g ^.^kg^-1^ BM per day) of Gly (Glycerin, Care plus, Huddersfield, UK) and 150 g/day (4 × 37.5 g/day) of Glu (SISGO Electrolyte Drink Powder, Ashwood Laboratories, Lancashire, England) and the participants of the Cr/Gly/Glu/Ala group to ingest 20 g/day (4 × 5 g/day) of Cr monohydrate (Creapure Creatine Monohydrate, Reflex Nutrition Ltd, UK), 2 g^.^kg^-1^ BM per day (x 0.5 g^.^kg^-1^ BM per day) of Gly (Glycerin, Care plus, Huddersfield, UK), 100 g/day (4 × 25 g/day) of Glu (SISGO Electrolyte Drink Powder, Ashwood Laboratories, Lancashire, England) and 1000 mg/day (4 × 250 mg/day) of Ala (Racemic mixture [R and S] Pure Bulk, USA) for 7 days. Both groups ingested the supplement assigned to them orally and were asked to consume four drinks per day. All supplements were made fresh before consumption to avoid degradation of Cr to creatinine. Participants were unlikely to recognize that Cr/Gly/Glu/Ala was less sweet as they were not aware of the sweetness of the Cr/Gly/Glu consumed by the other group. Participants in both groups started ingesting the final drink 5 h before performing the final trial (post supplementation exercise trial) with instruction to complete ingestion within 1 h. Commencement of ingestion of a hypertonic solution such as the Cr/Gly combination (965 ± 61 mOsm/kg) 5 h prior to exercise, has shown to result in a larger volume of fluid absorbed in comparison to ingestion 3 h prior to exercise [14]. Supplements in both groups had similar taste, texture and appearance and were placed in generic bottles to ensure double-blind administration [3]. On each of the experimental test days, participants ingested 1 L of water 3 h before exercise and a further 500 mL of water 1 h before exercise in an attempt to ensure that they were euhydrated before all exercise trials.

**Figure 1:**
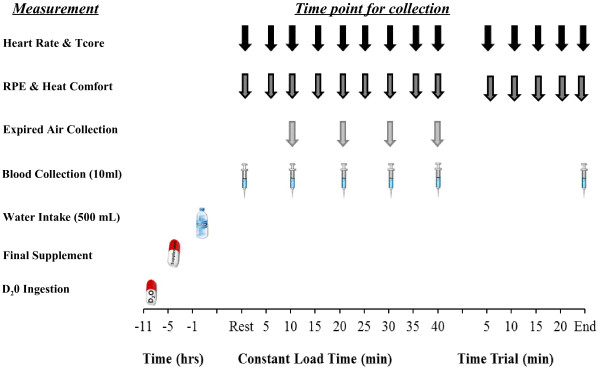
Schematic representation of experimental protocol.

All trials were separated by one week and the supplementation period for both groups started on the day after the 1st test and finished the day before the 2nd test. Participants in both groups were asked to consume 2 L of water per day during the familiarization week in order to standardize their fluid consumption and to allow for participants to act as their own controls. The pre and post supplementation trials also required participants to report to the laboratory before breakfast, after an 8 h fast, and ingest a small dose (1 g^.^kg^-1^ BM) of deuterium oxide (D_2_O) for the purpose of TBW determination. Each participant was also given an ingestible temperature sensor to swallow 8–12 h prior to each exercise trial [3]. In addition, during the morning trials, participants performed a re-breathing procedure, which involved the minimally invasive optimized carbon monoxide (CO)-rebreathing method as previously described [14-16]; a procedure that allowed for estimation of plasma and blood volume (PV) via the direct determination of total haemoglobin mass (tHb-mass). Participants were then free to leave the laboratory and were asked to return 11 h later (Figure 1) for the exercise trial. The 11 h gap between the morning and the exercise testing was introduced to allow for accurate TBW determination; the last urine sample required for determination of TBW with the D_2_O method was taken 6 h post D_2_O ingestion while the final supplement was ingested 5 h before the exercise trial but after the collection of the D_2_O urine sample, in order to avoid any possible acute effect of the hyper hydrating solution on TBW and interference with the D_2_O protocol.

Participants followed their normal diet and completed 7-day food diaries during the familiarization and pre supplementation weeks and were asked to replicate their training regimes throughout the study period. The diet was analyzed for energy intake and macronutrient content using the CompEat nutritional analysis software, which is based on the UK, integrated database, McCance and Widdowson’s [15]. Participants were asked to avoid caffeine intake and alcohol for the full length of their participation in the trial to lessen any possible confounding effects of caffeine on Cr [13].

### Experimental procedures: total body water determination

Participants were required to report to the laboratory before breakfast after an 8 h fast. Measurements of TBW using both BIA (Bodystat Multiscan 500, Bodystat Ltd, Isle of Man, UK) and D_2_O method were carried out. Briefly, BIA is an non-invasive method that involves placing two current-inducing electrodes and two detector electrodes on the dorsal surfaces of the right hand and foot and a small (and imperceptible) electrical current (500 Micro-Amps) introduced between these. On arrival to the laboratory, participants provided a baseline urine sample and were then asked to lie comfortably in a supine position while a 21 G cannula was introduced into a superficial vein on the dorsal surface of the participant’s arm. Blood samples (10 mL) were taken before and after the re-breathing procedure [16-18]. Participants were then asked to orally ingest D_2_O (Ontario hydro, Canada). The validity of method has been previously assessed [19]. Each participant was given an oral dose of 0.5 g^.^kg^-1^ BM of D_2_O in the morning after a baseline urine sample has been collected. To evaluate the volume of isotopic distribution in body water, a urine sample was collected again after 6 h, in a dry plastic container. Participants were instructed to empty their bladder completely at 5 h post D_2_O ingestion and were allowed breakfast, a light lunch as well as to pass urine and drink as normal within the 6 h period. For purposes of analysis, the investigator transferred 2 mL from all urine samples from the dry plastic containers to glass vessels and stored in −20°C. Urine samples were then analyzed by an isoprime isotope ratio mass spectrometer (Elementar Ltd, Manchester, UK), coupled to a Eurovector gas chromatograph (GC) fitted with an HT300A autosampler, as described elsewhere [20].

### Experimental procedures: Analyses of total haemoglobin mass

Briefly, a bolus of chemically pure CO dose of 1.0 mL^.^kg^-1^ BM was administered with the first breath through a spirometer and rebreathed for 2 min with 4 L of oxygen. The small individual non-toxic CO dose used for the rebreathing method may temporarily impair the oxygen transport capacity of blood and therefore muscle oxygen uptake. However, these effects are minimal and basal carboxyhaemoglobin concentrations will be achieved after 6 h [20]. There are no contraindications for the use of the rebreathing procedure after a competition or within training and recovery periods [20] and this method is considered less risky in participants performing maximal exercise. Change in percentage of carboxyhaemoglobin in venous blood samples (from baseline to 8 min after CO administration), analysed using a blood gas analyser (ABL 725, Radiometer, Copenhagen, Denmark), was used to determine tHb-mass. In addition, blood, erythrocyte and plasma volume were derived as previously described elsewhere [21].

### Experimental procedures: Exercise trials

When arriving to the laboratory after a 3 h fast, for the exercise trials, participants had their height and nude BM measured. Pre to post supplementation BM change determination acted as a supplementary indirect measurement of the volume of fluid retained. After the BM measurement, a venous cannula was inserted into an anticubital vein and a HR monitor (Polar Sports Tester, Polar Electro Oy, Kempele, Finland) was attached. Participants were then transferred to the climatic chamber (ambient temperature 30.0 ± 0.2°C with a relative humidity of 70% ± 0.3% and air velocity of 1.8 m/s) and seated on specialist cycle ergometer (HP Cosmos Cyclus 2 Record-trainer, Nussdorf-Traunstein, Germany) for 10 min as PV, a parameter of great interest; is known to be influenced by body posture [22].

Resting HR and Tcore were determined while the participant was seated on the cycle ergometer and a blood sample (10 mL) was obtained (Figure 1). The venous cannula was kept patent by a 10 mL infusion of isotonic saline between samples. Participants were then instructed to begin unloaded cycling for 5 min followed by a 40 min cycle at their predetermined WR (Cr/Gly/Glu group 277 ± 44 W, Cr/Gly/Glu/Ala group 242 ± 35 W). WR was increased in a ‘single step’ after the 5 min of unloaded cycling had been completed. Participants were required to maintain a pedal cadence of 70–100 revolutions/min throughout the 40 min constant load exercise. HR and Tcore were recorded every 5 min during the constant load exercise and time trial. Ratings of perceived exertion (RPE) were recorded at 5 min intervals of the 40 min constant-load exercise and time trial using the Borg category scale [21]. Additionally, heat comfort (HC) was determined using an adapted thermal comfort scale and recorded every 5 min during the 40 min constant load exercise and during the time trial [23]. Blood samples (10 mL) were obtained every 10 min during the constant load exercise and at the end of the time trial. An expired air collection was taken during the last minute of each 10 min stage using the Douglas bag technique [24]. Samples of expired air were collected using 150 L Douglas bag (Hans Rudolph 2100 three-way stop cock, Hans Rudolph Inc, Kansas City, USA) Participant, whilst wearing a nose clip, breathed through a mouthpiece fitted to a lightweight one way respiratory valve (2700 Series Large 2-way NRBV, Hans Rudolph Inc, Kansas City, USA), which in turn was connected to a 120 cm long lightweight tube with a diameter of 38 mm. The tubing terminated at a two-way valve which opened and closed the Douglas bag. A known volume (range between 200–350 ml/min) of expired air was extracted through the sampling port of the Douglas bag at a constant flow rate, controlled by a flow meter. This air passed into a gas analyzer (Servomex 1440 Gas Analyzer, Servomax Group Limited, East Sussex, England) to determine the percentage of oxygen (O_2_) and carbon dioxide (CO_2_). The remaining volume of expired air in each Douglas bag was measured by evacuation through a dry gas meter (Harvard Apparatus Inc, Holliston, USA). The temperature of the air in Douglas bag was measured during evacuation. The gas analyzer was calibrated before each sample analysis with nitrogen, a calibration gas (BOC Gases, BOC limited, Surrey, UK). Barometric pressure was recorded. The measured expired gas volumes were corrected to standard temperature and pressure for a dry gas using the universal gas equation. Inspired gas volume was derived using the Haldane transformation and used to calculate O_2_ and CO_2_, and RER as CO_2_/O_2_. Following the 40 min constant load exercise, the resistance was decreased to 10 W and participants were instructed to continue pedaling for an additional minute. The participant then commenced the 16.1 km (10 mile) self-paced time trial on the same cycle ergometer used in the constant load phase. Nude BM was measured post exercise and the difference before and after completion of exercise was used to estimate sweat loss and sweat rate. The time to completion of the time trial was recorder but only revealed to the participants upon completion of all trials.

### Blood treatment and analysis

In all trials, blood was drawn into dry syringes and 8 mL dispensed into two 4 mL tubes containing K_3_EDTA while the remaining 2 mL were dispensed into plain tubes. Duplicate aliquots (100 μL) of whole blood from the K_3_EDTA tube were rapidly deproteinized in 1000 μL of ice-cold 0.3-mol/L perchloric acid, centrifuged, and the supernatant used to measure Glu and lactate using standard enzymatic methods with spectrophotometer detection (Spectra Max M2 microplate reader). The remaining blood from the K_3_EDTA tube was analyzed for haemoglobin (cyanmethemoglobin method, Sigma, Chemical Company Ltd., Dorset, UK) and packed cell volume (conventional michrohematocrit method). The blood in the tube without anticoagulant was allowed to coagulate and then was centrifuged (8 min, 14,000 rpm, RT, Hettich Mikro 120); serum was collected and used to measure osmolarity by freezing point depression (Micro-osmometer 3300, Vitech Scientific, West Sussex, UK). Packed-cell volume was carried out in triplicate while all other blood analyses were carried out in duplicate. Changes in haemoglobin and packed-cell volume relative to initial baseline values were used to calculate PV changes during exercise [25].

### Statistical analysis

Data were assessed for normality of distribution and descriptive analysis was carried out to reveal the mean ± SD. Statistical analysis was carried out using the 3-factor mixed-model ANOVA with repeated measures, followed by a simple main effects analysis for significant 3-way interactions (i.e., pre vs. post supplementation at each time point and treatment), simple main effect analysis for 2-way interactions and post hoc analyses for any significant main effect detected within the model. In addition, paired or 2-samplet-tests were used to examine the magnitude of change (Δ) that occurred from the pre- to post-supplementation trials between the experimental groups (Cr/Gly/Glu and Cr/Gly/Glu/Ala), when difference was detected using the simple main effect analysis. Independent sample t-tests were used to examine pre supplementation differences between the two treatments. ANCOVA was carried out in cases where baseline differences were detected and pre supplementation values were used as covariates. All statistical analysis was carried out using SPSS for Windows version 17.0. Statistical significance was set at *P ≤* 0.05. Participants (one and two participants in Cr/Gly/Glu and Cr/Gly/Glu/Ala groups respectively) in whom TBW gain was < 0.2 L were considered as ‘non-responders’ and excluded from statistical analysis.

## Results

### Body mass and total body water

**Figure 2:**
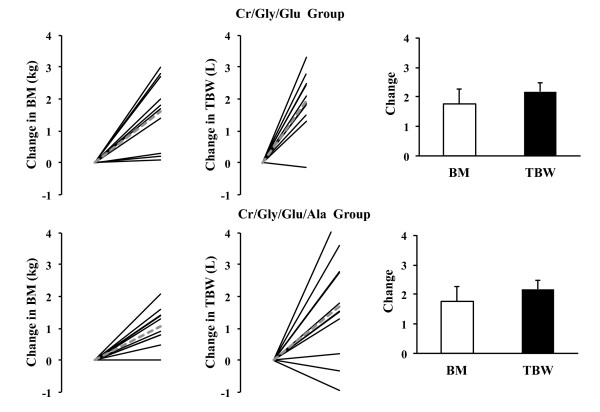
**Changes in Body Mass (BM) and Total Body Water (TBW) induced by supplementation in Cr/Gly/Glu (top) and Cr/Gly/Glu/Ala (bottom) groups.** Dashed lines represent mean of the group and solid black lines represent individual data (data presented for both ‘responders’ and ‘non-responders’). Bar charts represent Mean ± SD values of change in TBW and BM for responders only. *Significant (*P* < 0.05) difference between post and pre supplementation.

The physical characteristics of the groups were similar before supplementation (Figure 2). At baseline BM (*P* = 0.05) and TBW (*P* = 0.03) were significantly higher in the Cr/Gly/Glu/Ala than in the Cr/Gly/Glu group (Table 1). Baseline BM and TBW values were therefore used as covariates when examining the difference between groups in TBW change induced by supplementation. Measurements of TBW by D_2_O ingestion, which reflects responses to supplementation, identified that 3 participants (1 from Cr/Gly/Gly and 2 from Cr/Gly/Glu/Ala group) did not gain TBW. These participants were therefore excluded from statistical analysis. When analysis was carried out on responders, it was found that supplementation induced increase in TBW was significant in Cr/Gly/Gly and Cr/Gly/Glu/Ala groups (*P* = 0.03; Figure 2) and that increase in TBW was not different between two groups (*P* = 0.86). Changes in TBW measured by D_2_O ingestion and BIA, were not significantly correlated (*P* = 0.40; *r =* 0.20). Change in BM after supplementation (*P* = 0.75) was not significant in any of the groups (Figure 2). Correlation between changes in BM and TBW was not significant (*P* = 0.06; *r =*0.40).

### Cardiopulmonary variables

**Figure 3:**
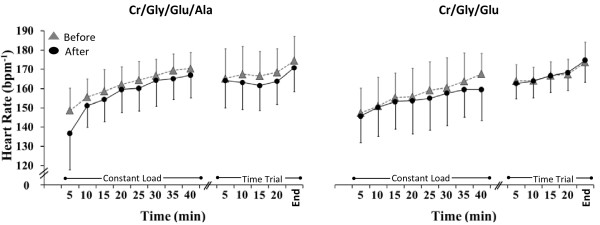
**Heart rate (HR) during exercise before (grey triangles) and after (black circles) supplementation in the Cr/Gly/Glu/Ala and Cr/Gly/Glu groups.** Data presented as Mean ± SD. *(*P* = 0.01) for significant difference between after and before supplementation.

There was no significant change in O_2_ or CO_2_ during constant-load exercise, and no differences were found between groups before or after supplementation (Table 2). RER, on the other hand, was significantly overall higher post compared to pre supplementation in the Cr/Gly/Glu group (*P =* 0.01) but not in the Cr/Gly/Glu group (Table 2). A significant 3- or 2-way interaction for heart rate (HR) was not found, thus the main effects were interpreted. During exercise, HR increased significantly over time (*P* = 0.01). Overall, HR was significantly lower post supplementation (*P* = 0.39) (Figure 3). In pre supplementation trials HR during exercise was not significantly different between the 2 groups.

**Table 2:** Cardiopulmonary responses throughout exercise

**Variable**		**Time (min)**				
		**Trial**	**10**	**20**	**30**	**40**
O_2_ (ml/kg/min)	Cr/Gly/Glu	Pre	42.9 ± 6.1	43.1 ± 7.4	44.2 ± 6.2	44.6 ± 7.3
		Post	42.2 ± 6.7	42.1 ± 6.6	40.8 ± 6.4	42.3 ± 6.2
	Cr/Gly/Glu/Ala	Pre	40.9 ± 4.8	41.9 ± 5.1	42.7 ± 4.8	42.3 ± 5.2
		Post	41.8 ± 3.4	41.5 ± 2.9	41.8 ± 4.1	42.3 ± 3.7
CO_2_ (ml/kg/min)	Cr/Gly/Glu	Pre	41.5 ± 6.1	41.0 ± 7.4	41.7 ± 4.9	41.8 ± 7.6
		Post	41.4 ± 4.7	42.0 ± 4.8	42.0 ± 4.6	42.1 ± 5.1
	Cr/Gly/Glu/Ala	Pre	42.3 ± 7.2	41.2 ± 7.3	39.9 ± 6.7	41.2 ± 6.6
		Post	41.2 ± 3.1	41.0 ± 3.5	41.2 ± 3.5	41.3 ± 3.9
RER	Cr/Gly/Glu	Pre	0.94 ± 0.0	0.94 ± 0.0	0.94 ± 0.1	0.93 ± 0.0
		Post*	0.98 ± 0.0	0.97 ± 0.0	0.97 ± 0.0	0.97 ± 0.0
	Cr/Gly/Glu/Ala	Pre	0.98 ± 0.0	0.98 ± 0.0	0.96 ± 0.0	0.97 ± 0.0
		Post	0.97 ± 0.0	0.97 ± 0.0	0.97 ± 0.0	0.96 ± 0.0

Oxygen consumption (O_2_) and carbon dioxide production (CO_2_), and respiratory exchange ratio (RER) in Cr/Gly/Glu and Cr/Gly/Glu/Ala groups during exercise before and after supplementation. Data presented as Mean ± SD.

### Core temperature (tcore) responses

**Figure 4:**
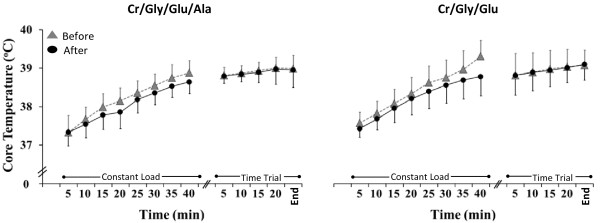
**Core temperature (Tcore) during exercise before (grey triangles) and after (black circles) supplementation in the Cr/Gly/Glu/Ala and Cr/Gly/Glu groups.** Data presented as Mean ± SD. *(*P* = 0.01) for significant difference between after and before supplementation.

Pre supplementation Tcore was similar in the 2 groups of participants (*P >* 0.05). A significant 3- or 2-way interaction was absent for Tcore; hence the interpretation of the main effects. Throughout the exercise period, Tcore increased significantly (*P* = 0.01; Figure 4). Overall, Tcore was significantly lower during exercise conducted after supplementation (*P* = 0.01).

### Ratings of perceived exertion (RPE) and heat comfort (HC)

**Figure 5:**
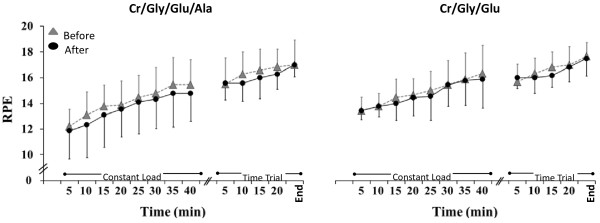
**Rate of perceived exertion (RPE) during exercise before (grey triangles) and after (black circles) supplementation in the Cr/Gly/Glu/Ala and Cr/Gly/Glu groups.** Data presented as Mean ± SD.

**Figure 6:**
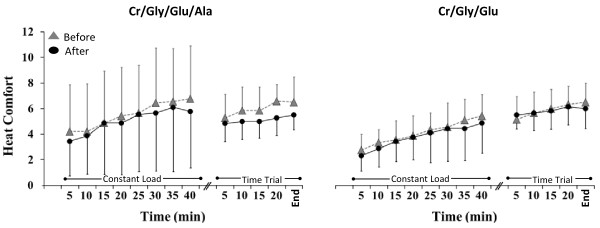
**Heat comfort (HC) during exercise before (grey triangles) and after (black circles) supplementation in the Cr/Gly/Glu/Ala and Cr/Gly/Glu groups.** Data presented as Mean ± SD.

No significant 3- or 2-way interactions were obtained for RPE and HC, thus the main effects were interpreted. Over time, RPE (Figure 5) increased significantly during all exercise trials (*P* = 0.01) but no significant differences were found in RPE between and after supplementation (*P =* 0.53). Similarly, HC increased significantly throughout exercise in all trial over time during all exercise trials *(P =* 0.01) but no significant differences were found in HC between and after supplementation *(P =* 0.69; Figure 6).

### Urine osmolality

No significant changes were found between pre (Cr/Gly/Glu, 147 ± 60 mOsm/L Cr/Gly/Glu/Ala, 172 ± 66 mOsm/L) and post (Cr/Gly/Glu, 182 ± 70 mOsm/L; Cr/Gly/Glu/Ala, 249 ± 171 mOsm/L) supplementation in urine osmolality (*P* = 0.06).

### Sweat loss and sweat rate during exercise

Sweat loss during exercise was not significantly different between groups in the pre supplementation phase. In both groups supplementation induced no change in sweat loss (Cr/Gly/Glu group, Pre: 1188 ± 434 ml, Post: 1277 ± 307 ml; Cr/Gly/Glu/Ala group, Pre: 1477 ± 569 ml, Post: 1600 ± 371 ml; *P* = 0.47).

### Blood metabolites

Resting blood lactate concentration was not significantly different between pre and post supplementation in either of the supplementation groups (*P* = 0.41; Table 3) and thus supplementation-induced changes were not different between groups. Blood lactate concentration increased throughout exercise in all trials but supplementation had no effect on overall mean lactate concentration changes during constant load exercise (*P =* 0.71) or on lactate values at the end of the time trial (*P* = 0.10) and no difference was found between groups. No significant difference was found in resting blood Glu concentration in Cr/Gly/Glu and Cr/Gly/Glu/Ala between pre and post supplementation trials (*P* = 0.97; Table 3) and supplementation-induced changes were not different between the groups. Glu concentration values during constant load exercise and Glu values at the end of the time trial were not affected by supplementation and thus supplementation-induced changes were not different between groups (Constant load Glu concentration (pre vs. post): *P* = 0.89; Time trial Glu concentration (pre vs. post): *P* = 0.92).

**Table 3:** Blood metabolite changes at rest and throughout exercise

**Variable**		**Time (min)**			
		**Trial**	**Rest**	**During**	**End**
Lactate (mmol/L)	Cr/Gly/Glu	Pre	0.9 ± 0.3	4.1 ± 0.2	6.2 ± 2.5
		Post	1.1 ± 0.3	5.1 ± 0.5	8.5 ± 2.7
	Cr/Gly/Glu/Ala	Pre	0.9 ± 0.2	4.5 ± 0.3	5.2 ± 1.6
		Post	1.3 ± 1.1	4.9 ± 0.5	7.1 ± 2.6
Glucose (mmol/L)	Cr/Gly/Glu	Pre	4.9 ± 0.3	5.4 ± 0.6	5.4 ± 0.6
		Post	4.9 ± 0.3	5.3 ± 0.7	5.3 ± 1.2
	Cr/Gly/Glu/Ala	Pre	4.9 ± 0.4	4.5 ± 0.2	4.4 ± 0.9
		Post	4.9 ± 0.2	4.6 ± 0.1	4.6 ± 0.7

Blood metabolite changes at rest, throughout exercise and at the end of the time trial in Cr/Gly/Glu and Cr/Gly/Glu/Ala groups during exercise before and after supplementation. Data presented as Mean ± SD.

### Plasma volume changes and total hemoglobin mass

No significant differences were observed between the pre- and post-supplementation phase for tHb-mass (Cr/Gly/Glu, Pre: 951 ± 93 g, Post: 949 ± 85 g; Cr/Gly/Glu/Ala, Pre: 1086 ± 172 g, Post: 1066 ± 164; g *P* = 0.96). PV change was reduced approximately by 15% and by 8% during exercise in the pre and post supplementation trials respectively, of the Cr/Gly/Glu group and by 13% and 12% in the pre- and post-supplementation trials respectively, of the Cr/Gly/Glu/Ala group. Supplementation had no effect on PV decrease during exercise and thus supplementation induces changes were not different between the groups. Additionally, PV estimated with the use of the optimized CO-monoxide rebreathing method was not significantly different pre- to post-supplementation (Cr/Gly/Glu, Pre: 4246 ± 424 mL, Post: 4274 ± 458 mL; Cr/Gly/Glu/Ala, Pre: 4698 ± 471 mL, Post: 4830 ± 571 mL; *P* = 0.62).

### Osmolality

Resting serum osmolality did not differ between pre (284 ± 19 mOsm·kg^-1^) and post supplementation (283 ± 18 mOsm·kg^-1^) in the Cr/Gly/Glu group and pre (277 ± 33 mOsm·kg^-1^) and post supplementation (284 ± 18 mOsm·kg^-1^) in the Cr/Gly/Glu/Ala group. Additionally, no significant differences were found in serum osmolality over time during the exercise trials, between (*P* = 0.83) or within treatments (*P* = 0.29).

### Time-trial performance

Before supplementation time-trial performance was not significantly different (*P* = 0.62) between the groups. Time-trial performance was not significantly influenced by supplementation (*P* = 0.75) in either the Cr/Gly/Glu group (Pre, 26:47 ± 1:09 min, Post, 26:25 ± 1:06 min) or the Cr/Gly/Glu/Ala group (Pre, 27:12 ± 2:04 min, Post, 26:53 ± 2:06 min).

### Energy and macronutrient intake

In both groups during week preceding supplementation (Pre) and supplementation week (Sup) averaged daily energy intake (Cr/Gly/Glu group, Pre: 2489 ± 498 Kcal, Sup: 1959 ± 251 Kcal; Cr/Gly/Glu/Ala group, Pre: 2571 ± 220 Kcal, Sup: 2048 ± 391 Kcal) was significantly lower (*P* < 0.01). Averaged available CHO intake (Cr/Gly/Glu group, Pre: 470 ± 114 g, Sup: 612 ± 46 g; Cr/Gly/Glu/Ala group, Pre: 376 ± 247 g, Sup: 595 ± 247 g) was significantly higher (*P* < 0.01), averaged fat intake (Cr/Gly/Glu group, Pre: 103 ± 38 g, Sup: 63 ± 10 g; Cr/Gly/Glu/Ala group, Pre: 101 ± 28 g, Sup: 83 ± 25 g) lower (*P* < 0.01) and averaged protein intake (Cr/Gly/Glu group, Pre: 86 ± 16 g, Sup: 99 ± 12 g; Cr/Gly/Glu/Ala group, Pre: 114 ± 29 g, Sup: 112 ± 31 g) did not differ between pre and during supplementation period (*P* = 0.49).

### Side effects

In general, participants tolerated the supplementation protocol well, with only 1 report of gastrointestinal distress after Cr/Gly/Glu supplementation that withdrew from the experimental process before completing the post supplementation trial. All participants from both groups were unsure of the treatment they received.

## Discussion

This is the first study to compare the thermoregulatory, cardiovascular and exercise performance effects during exercise in the heat induced by a known hyper hydrating supplement comprising of Cr/Gly and Glu [3,4] and a newly designed supplement. The newly designed supplement differs from the already tested Cr/Gly/Glu, in the fact that part of the Glu is replaced by Ala. Ala is a compound characterized by the pronounced insulin-potentiating activity and thus known to potentiate Cr uptake under conditions when amount of carbohydrate added is significantly lower than the doses recommended for hyper hydrating supplement of Cr/Gly/Glu [10]. The main finding of this study is that improvements in thermoregulatory and cardiovascular responses during exercise in the heat induced by Cr/Gly supplement containing excessive amounts of Glu and by Cr/Gly supplement containing Ala and lower amount of Glu are similar. We also found that exercise performance measured as time required to cover 16.1 km distance by cycling at 30.0°C and relative humidity of 70% was not improved following consumption of both supplements.

Ability of Cr/Gly/Glu and Cr/Gly/Glu Ala supplements to attenuate increase in Tcore and HR during exercise in the heat to a similar extent is not surprising, since in TBW increase in both groups was very similar and equal to 1.7 ± 1.1 and 1.2 ± 0.5 L in Cr/Gly/Glu and Cr/Gly/Glu Ala, respectively. The current study identified that following supplementation TBW was unchanged in 17% of participants; one from Cr/Gly/Gly group and two from Cr/Gly/Glu/Ala group. This most likely indicates that in these three participants Cr uptake was negligible and not sufficient for fluid retention in intracellular fluid compartments [5]. Therefore these participants were considered as ‘non-responders’ and excluded from statistical analysis. This decision was made on previous suggestion that failure to discriminate between those who respond to Cr supplementation and those who do not could mask any effect resulting from Cr supplementation [5]. No response to Cr supplementation by some participants is not surprising since muscle biopsies studies measuring Cr concentration before and after supplementation found that approximately 20–25% of the population show very little or no response to Cr supplementation [26]. This can be explained by the fact that uptake of Cr by the skeletal muscle is very much dependent on initial Cr pool with uptake being highest in those with low levels [27]. Although Cr uptake has been previously estimated with the measurement of urinary creatinine [5], this method requires the collection of urine for the duration of the supplementation period (7- days in the case of this study), which would be impractical, and too demanding for the participants of the current study. Previous study by Powers et al. (2003), which used muscle biopsy technique for the measurement of Cr uptake and D_2_O method for the measurement of TBW, has shown that increase in TBW was directly associated with Cr uptake [28].

In most previous studies examining the effects of Cr/Gly supplementation on hyper hydration, response to Cr/Gly supplement was determined by considering changes in BM rather than TBW changes [3,4]. In our study both supplementation did not induce significant increase in BM, which is different to previous studies [3,4]. It should be noted that changes in BM are influenced not only by hyper hydrating substances but also by changes in energy intake and energy expenditure during days of supplementation. In our study, during the week of supplementation energy intake including energy obtained from supplements was significantly lower. In addition some participants reported an ability to work harder in the training sessions during week of supplementation. Therefore, hyper hydration induced increase in TBW may not necessarily be reflected in BM. Gold standard technique such as D_2_O ingestion, for TBW measurements should be considered, since our study also demonstrated that correlation between TBW changes measured by D_2_O ingestion and estimated by BIA was not significant.

Another aspect related to the increase in TBW and is worth discussing, is the implication of TBW increase on PV. This was the first study to estimate impact of supplementation on pre exercise PV, via the direct measurement of tHb-mass with the use of the optimized CO-monoxide method [18]. Both supplementations had no significant impact on PV although TBW increased by 0.2 – 4.6 L. We note that in our study estimated PV change following supplementation was small in relation to total PV and consisted of 28 mL and 132 mL in Cr/Gly/Glu and Cr/Gly/Glu/Ala groups, respectively, which is in accordance with suggestion of Latzka et al. (1998) [29]. It is unlikely that a PV increase between 28–132 mL as occurred in the current study, accounts for the attenuation in the rise in Tcore and HR. Indeed, in studies where substantial alterations in cardiovascular function and heat storage by PV expansion were recorded, the magnitude of the PV changes was large (300–700 ml) [30-33].

Extend of supplementation induced attenuation of the increase in Tcore and HR during exercise seen in our study, is in consistency with previous studies [3,4]. Rise in Tcore was reduced by 0.2 and 0.3°C following Cr/Gly/Glu and Cr/Gly/Glu/Ala supplementation respectively (Figure 4). Hyper hydration achieved through Cr/Gly/Glu and Cr/Gly/Glu/Ala supplementation in the present study was also successful in attenuating the increase in HR by up to 2 and 4 beats/min respectively, during the constant load exercise in the heat (Figure 3). This was achieved regardless of the fact that changes in PV during exercise conducted before and following supplementations were not significantly different. Therefore, it seems that improvement in thermoregulation induced by hyper hydration strategies used in this study were achieved by PV and sweat rate maintenance [34] and by increasing the specific heat capacity of the body as suggested by Easton et al. (2007) and Beis et al. (2011), rather than PV expansion.

We found that in Cr/Gly/Glu group, following supplementation, RER during constant load exercise was significantly higher than in the pre supplementation trial which reflects the contribution of CHO towards energy production being enhanced and contribution of fat reduced by consumption of the Cr/Gly/Glu supplement. This finding is not surprising since daily amount of Glu consumed with the Cr/Gly/Glu supplement for the duration of seven days was as high as 150 g and significantly increased intake of available CHO. It is well established that increased dietary carbohydrate intake for several days increases muscle glycogen concentration [35,36] and that energy substrate selection during exercise to a great degree depends on muscle glycogen availability [37,38]. In Cr/Gly/Glu/Ala group, RER values measured during constant load exercise were not significantly different between pre and post supplementation trials. This can be explained by lower intake of Glu within the Cr/Gly/Glu/Ala supplement in comparison to the Glu contained in the Cr/Gly/Glu supplement. Regardless of the possible enhanced availability of muscle glycogen and change in energy substrate utilization during exercise following Cr/Gly/Glu suplement, it is unlikely that this could have impact on exercise performance due to muscle glycogen depletion. This suggestion receives support from no hypoglycemia being sees at point of completion of all time trials.

Despite the decrease in Tcore and HR during constant load exercise experienced by both supplementation groups in the present study, time trial performance was not affected which is in consistency with some hyper hydration studies [3,39,40] but contradict findings of other researchers [5,41-43]. It should be noted, that studies finding a positive effect of hyper hydration on exercise performance, employed protocols different from that in our study. For example, in the study by Anderson et al. (2001), participants were required to cycle for 90 min at a constant load before commencing the time trial. This duration is more than twice the duration employed in the current study. In addition, it might be that in our study, intensity of constant load exercise has not been high enough since mean values of RPE were 15 and 14 in Cr/Gly/Glu and Cr/Gly/Glu/Ala group, respectively (Figure 5). It is therefore possible, that the exercise trial in the present study was not of sufficient duration and intensity for hyper hydration to have a significant effect on performance. On the other hand, values for mean O_2_ during constant load exercise were 44 and 42 ml/kg/min in Cr/Gly/Glu and Cr/Gly/Glu/Ala group, respectively, which consisted of ~ 70% of O_2_max (Table 1) and should be high enough to induce fatigue prior to the time trial. It is therefore possible that commencing exercise in a hyper hydrated state might not confer any significant advantage in terms of exercise performance as found in the studies by Easton et al. (2007), Marino et al. (2003), and Latzka et al. (2000). In either case, studies with duration and conditions sufficient to induce a higher degree of dehydration should be carried out to examine whether hyper hydration can have a significant effect on exercise performance.

## Conclusion

In comparison to the established hyper hydrating Cr/Gly/Glu supplement, supplement containing Cr/Gly/Ala and decreased amount of Glu provides equal improvements in thermoregulatory and cardiovascular responses during exercise in the heat. Nevertheless, administration of both supplements had no effect on exercise performance.

## Competing interests

The authors declare that they have no competing interests.

## Authors’ contributions

TPP assisted in the design of the study, participant recruitment, study management, data collection and analysis and was the primary author of the manuscript. TP was involved in participant recruitment, data collection and analysis. DM assisted in study supervision and coordination and was involved in data analysis and editing the manuscript. TP and WCL were involved in participant recruitment, data collection and analysis. JRS and CH participated sample analysis and manuscript editing. YPP conceived of the study, participated in its design and coordination and helped to draft the manuscript. All authors read and approved the final manuscript.
